# Abdominal aortic calcification quantified by the Morphological Atherosclerotic Calcification Distribution (MACD) index is associated with features of the metabolic syndrome

**DOI:** 10.1186/1471-2261-11-75

**Published:** 2011-12-20

**Authors:** Natasha Barascuk, Melanie Ganz, Mads Nielsen, Thomas C Register, Lars M Rasmussen, Morten A Karsdal, Claus Christiansen

**Affiliations:** 1 Nordic Bioscience A/S, Herlev Hovedgade 207, 2730 Herlev, Denmark; 2 Department of Computer Sciences, University of Copenhagen, Universitetsparken 1, 2100 Copenhagen Ø, Denmark; 3 Department of Physiology, Section on Comparative Medicine, Wake Forest University Medical School, NC, USA; 4 Department of Clinical Biochemistry, Odense University Hospital, University of Southern Denmark, 5000 Odense, Denmark

**Keywords:** Cardiovascular disease, aortic calcification, risk factors, AAC24

## Abstract

**Background:**

Abdominal aortic calcifications (AAC) predict cardiovascular mortality. A new scoring model for AAC, the Morphological Atherosclerotic Calcification Distribution (MACD) index may contribute with additional information to the commonly used Aortic Calcification Severity (AC24) score, when predicting death from cardiovascular disease (CVD). In this study we investigated associations of MACD and AC24 with traditional metabolic-syndrome associated risk factors at baseline and after 8.3 years follow-up, to identify biological parameters that may account for the differential performance of these indices.

**Methods:**

Three hundred and eight healthy women aged 48 to 76 years, were followed for 8.3 ± 0.3 years. AAC was quantified using lumbar radiographs. Baseline data included age, weight, blood pressure, blood lipids, and glucose levels. Pearson correlation coefficients were used to test for relationships.

**Results:**

At baseline and across all patients, MACD correlated with blood glucose (r^2 ^= 0.1, P< 0.001) and to a lesser, but significant extent with traditional risk factors (p < 0.01) of CVD. In the longitudinal analysis of correlations between baseline biological parameters and the follow-up calcification assessment using radiographs we found LDL-cholesterol, HDL/LDL, and the ApoB/ApoA ratio significantly associated with the MACD (P< 0.01). In a subset of patients presenting with calcification at both baseline and at follow-up, all cholesterol levels were significantly associated with the MACD (P< 0.01) index. AC24 index was not correlated with blood parameters.

**Conclusion:**

Patterns of calcification identified by the MACD, but not the AC24 index, appear to contain useful biological information perhaps explaining part of the improved identification of risk of cardiovascular death of the MACD index. Correlations of MACD but not the AC24 with glucose levels at baseline suggest that hyperglycemia may contribute to unique patterns of calcification indicated by the MACD.

## Background

Cardiovascular disease (CVD) remains the leading cause of mortality and major cause of morbidity worldwide, with underlying atherosclerosis as one of the key elements. In more than 90% of cases, the cause of myocardial ischemia is atherosclerotic plaque progression and rupture which leads to thrombus formation and obstruction of blood flow in the coronary arteries.

More than 50% of patients die without clinical symptoms [1]. To identify asymptomatic individuals at highest risk, attempts have been made on developing tools to risk-stratify individuals with sub-clinical atherosclerosis.

Many risk factors of atherosclerosis have been identified, of which the most widely accepted are elevated total and low-density lipoprotein (LDL) cholesterol, reduced high-density lipoprotein (HDL) cholesterol, hypertension, obesity, diabetes, and cigarette smoking [2-4]. In the face of these well-recognized risk factors, it is still debated whether postprandial serum triglyceride levels are an independent predictor of cardiovascular risk. Some studies have indicated that non-fasting triglyceride levels are associated with incident of cardiovascular events independent of the traditional risk factors whereas fasting triglyceride levels have displayed little independent relationship [5,6]. In contrast, we have demonstrated that fasting triglyceride levels in combination with specific distributions of adiposity are strong predictors of accelerated atherosclerosis [7-9]. These and other discrepancies highlight the need for additional understanding of risk factors of CVD.

Non-invasive diagnostic tools, such as imaging techniques, for identification of advanced atherosclerotic plaques prior to manifestation of clinical symptoms, or even death, would be clinically useful. Calcification of coronary arteries has been shown to be directly related to the severity and the extent of underlying coronary plaque burden [10,11], and to be associated with increased risk of coronary heart disease (CHD) [12-14].

A variety of imaging techniques for examining vascular calcifications have emerged with increasing attention being focused on the assessment of abdominal aortic calcifications from simple X-rays. Lumbar abdominal X-ray for investigation of lumbar aortic calcification has been shown to correlate with the extent of calcified plaques in the coronary arteries [15-17]. These calcifications may be important risk factors of congestive heart failure (CHF) independent of CHD and other risk factors.

X-rays are traditionally analysed according to the categorical Framingham score system. We have recently demonstrated [18,19] that additional information could be harvested from these x-rays by including measures of the number, length, width, and morphology of the calcified plaques. Assessment of risk of CVD death using this novel quantification system Morphological Atherosclerotic Calcification Distribution (MACD) quantification system resulted in an odds ratio of 20 compared with 4.5 using the Framingham scoring systems [20]. Interestingly, the MACD was associated with CVD death independently of traditional risk factors including BMI, hypertension, obesity etc, suggesting that the MACD index contained additional critically useful information.

The aim of the current study was to further investigate the biological basis for the predictability of the MACD index. In post-menopausal women as previously described [18,19], we correlated traditional risk factors with MACD scores in a cross-sectional and a longitudinal study, and compared these correlations to the gold standard, the AC24 index developed on the basis of the Framingham Heart Study cohorts.

## Methods

### Subjects and Demographics

In 1992-93, 686 postmenopausal women living in the Copenhagen area were recruited via questionnaire surveys to participate in a study examining the role of a number of metabolic risk factors in the pathogenesis of CVD and osteoporosis. Follow-up was performed after 8.3 ± 0.3 years. Information from the 95 individuals who died in the observation period was obtained via the Central Registry of the Danish Ministry of Health with a follow-up rate of 100%. A total of 129 women had relocated from the Copenhagen area or did not wish to participate in the follow-up period and provided no clinical data at the end of the study. Baseline demographics and risk parameters were not different between the women who died or completed the study.

Among the 462 women completing the follow-up visit, 256 (55%) had radiographs in which the full lumbar (L1-L4) aorta was visible on a single radiograph. Furthermore, among the deceased, 52 (55%) had X-ray examinations in which the lumbar aorta was visible on a single radiograph. Of these 52 deaths, 20 were related to CVD (38%), 27 were related to cancer (52%) and 5 were related to other causes (10%). The total number of patients included in the final data set is 308.

All participants gave informed consent to participate and the study was carried out according to the Helsinki Declaration II and the European Standards of Good Clinical Practice. Local ethical committees have approved the study protocols.

At baseline and follow-up, data on age, weight, height, Body Mass Index (BMI), waist and hip circumferences, systolic and diastolic blood pressure (BP), and smoking were collected. Fasting plasma glucose and the lipid profile (total cholesterol, triglycerides, LDL-cholesterol (LDL-C), HDL-cholesterol (HDL-C), apolipoprotein ratio (ApoB/ApoB)) were obtained using an auto-analyzer (Cobas Mira Plus, Roche Diagnostics Systems, Hoffman-La Roche).

#### Assessment method of Abdominal Aortic Calcifications

Lateral X-rays of the lumbar aorta of all study participants (L1-L4) were recorded. The images were digitized using a Vidar Dosimetry Pro Advantage scanner providing an image resolution of 9651 by 4008 pixels on 12-bit gray scale using a pixel size of 44.6 μm^2^. Trained radiologists were instructed to annotate the 6 points used for vertebral height measurements [21] in the lumbar region L1- L4 on the digitized images, to delineate the aorta, and to outline every individual calcified deposit visible in the lumbar aorta while noting any association to anterior and/or posterior aortic walls. These readings recorded on a Sectra radiological reading unit with annotation software implemented in Matlab (Mathworks, MA, USA), allowed for the automatic computation of both the 0-24 aortic calcifications (AC24) severity score and the MACD [18]. The software used allowed editing and digital zoom.

The inter- and intra-observer variability of the radiologist's outlines of the AAC's was computed on a subset of 8 images with four annotations each. The mean Jaccard Index between the radiologists' AAC outlines was 0.56 for the intra-observer variation and 0.51 for the inter-observer variation. The two radiologists had an intra-observer variability of 0.53 and 0.59, respectively.

A newly developed aortic calcification index, MACD [18] was quantified from outlines of the calcified aortic plaques on the X-rays from the lumbar aorta at baseline and compared with scores derived from the same patients using the aortic calcification severity score, AC24. MACD is given by

**Figure 1:**
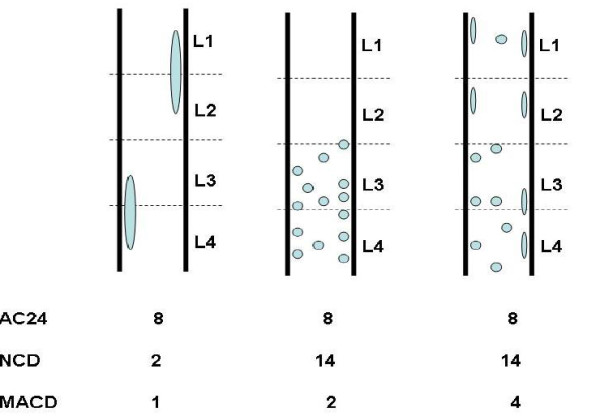
**Examples of scores for the AC24 and MACD index according to the size and distribution of the aortic calcifications**. Only MACD is influenced by both size and distribution of the lesions.

MACD=NCD⋅MAD,

where *NCD *is the Number of Calcified Deposits and given by the number of distinct calcified deposits in the lumbar region (L1-L4), as visualised in Figure 1. *MAD *is the Morphological Atherosclerotic Distribution factor and can be explained in the following way: X-ray analysis only visualises the calcified core and not the biological extend of the atherosclerotic lesions. As a consequence, we estimate the atherosclerotic plaque size from the area and form of the observed calcified lesion. This is done by quantization of not only the calcified part, but also pixels within a predefined distance of the calcification. Elongated and circular plaques of similar size do not count equally: the estimated plaque around the elongated calcification will be larger than the estimated plaque around the circular calcification of similar size, as visualised in Figure 1. Thus, equal areas of calcification with different morphologies will have different scores. The MAD factor is hence described mathematically by

$$MAD=\frac{sim}{area},$$

where *sim *is the simulated area described below and *area *is the area of the calcified plaque visible on the radiograph. The simulated area is implemented by iterated morphological dilations [18] with a combined radius of 200 pixels (corresponding to 8.9 mm) simulating the total extent of the atherosclerotic process. The radius of 200 pixels of the increment was found after experiments with about 30 images, which are a subset of our population. In summary, the MAD factor describes the growth potential of the calcified plaques.

#### Statistical Evaluation

Data is expressed as means ± SEM unless otherwise indicated. For analysis of the relation between metabolic markers and calcification markers the calcified population is divided into quartiles according to the calcification marker. As AC24 is categorical, patients are divided into quartiles Q1-Q4 according to their AC24 scores and into bins of similar size using the continuous MACD. Preliminary inspection showed that the highest quartile (Q4) stands out in biomarker values compared to Q1-Q3. Hence, we tested difference between Q4 and Q1 and also Q4 to Q1-Q3, where we pooled quartiles to get larger groups and drive significance. Differences to Q1 were never significant. All concentrations were log-transformed and visually inspected for normality. Differences were tested with two-sided t-tests. Similarly, patients were divided according to standard clinical thresholds for the metabolic markers and the difference in calcification markers in the two groups was tested with a non-parametric Mann-Whitney U test since calcification markers cannot be assumed to be normally distributed. Tests were considered significant when p < 0.05.

## Results

**Figure 2:**
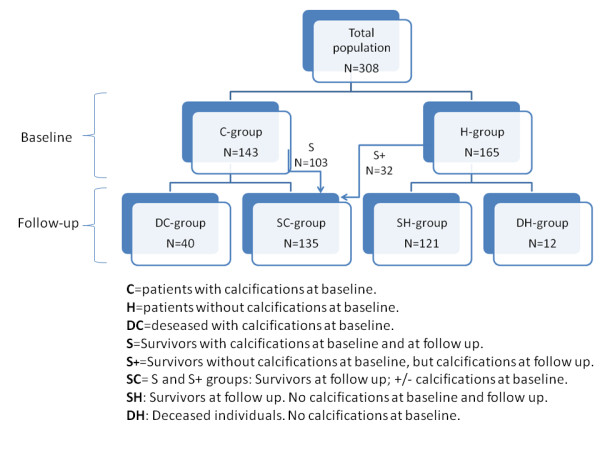
**Study population and the sub-populations**.

Baseline characteristics of the biochemical parameters for the 308 women who participated in this study are shown in Table 1. The mean age of the total population at baseline was approximately 60 years, with individuals with AACs at baseline having a significantly higher average age. There was a significant difference in systolic blood pressure, total cholesterol, LDL cholesterol, ApoB/ApoA, triglycerides, glucose, and BMI between the subgroup of patients (n = 165) with no calcifications at baseline, and the subgroup of patients (n = 143) presenting with aortic calcifications at baseline. Figure 2 illustrates the population subgroups at baseline and follow-up visits. Furthermore, Table 2 illustrates the calcification status of the study population at baseline and at follow up.

**Table 1:** Characteristics of the study population stratified into whole population, healthy (H) at baseline, with no calcifications identified, and population with calcifications (C) at baseline

	Population (n = 308)	H (n = 165)	C (n = 143)	
**Age (years)**	60.3 ± 7.5	57.8 ± 6.8	63.3 ± 7.1	**
**BMI (kg/m^2^)**	24.7 ± 3.9	25.1 ± 3.8	24.3 ± 4.0	
**Systolic BP (mm Hg)**	127 ± 21	122 ± 19	133 ± 23	**
**Diastolic BP (mm Hg)**	77 ± 10	76 ± 10	77 ± 10	
**Hypertension**	48	16	32	
**Glucose (mmol/L)**	4.74 ± 1.10	4.59 ± 0.56	4.92 ± 1.44	*
**Total-C. (mmol/L)**	6.33 ± 1.19	6.11 ± 1.00	6.58 ± 1.27	**
**Triglyceride (mmol/L)**	1.24 ± 0.75	1.20 ± 0.69	1.41 ± 0.81	*
**LDL-C/mmol/L)**	2.89 ± 0.82	2.71 ± 0.69	3.09 ± 0.91	**
**HDL-C/mmol/L)**	1.37 ± 0.36	1.38 ± 0.34	1.35 ± 0.39	
**ApoB/ApoA**	0.57 ± 0.18	0.53 ± 0.15	0.61 ± 0.20	**
**LDL/HDL**	2.32 ± 1.08	2.14 ± 0.92	2.53 ± 1.21	

* p < 0.05; ** p < 0.01; *** p < 0.001; (*) p < 0.1 borderline significant

**Table 2:** Overview of the groups in our study population according to patient calcification status at baseline and follow-up

Calcification Status	No calcification at Follow-up	Calcifications at Follow-up	Death at Follow-up
**Calcifications at Baseline**	-	S (n = 103)	DC (n = 40)
**No Calcifications at Baseline**	SH (n = 121)	S+ (n = 32)	DH (n = 12)

### Cross-sectional analysis

The MACD index was compared to the AC24 score with the respect to their respective relation to biological parameters. Differences between the highest quartile of calcified patients and the remainder of calcified patient (group C) are shown in Table 3.

**Table 3:** Cross-sectional analysis - division of different biological risk factors into quartiles based on AC24; Here presented mean plus/minus standard error of the mean and results of a two-sample t-test between the highest quartile and the combination of the lowest three quartiles with baseline biochemistry and baseline X-rays

Baseline-Baseline	MACD Group C Lowest three quartiles n = 116	MACD Group C Highest quartile n = 27	MACD Group C p-value	AC24 Group C Lowest three quartiles n = 116	AC24 Group C Highest quartile n = 27	AC24 Group C p-value
**Age**	63.08 ± 0.66	64.24 ± 1.39	-	62.84 ± 0.67	65.28 ± 1.25	-

**Triglyceride**	1.28 ± 0.07	1.95 ± 0.20	p = .0002	1.35 ± 0.07	1.65 ± 0.19	-

**BMI**	24.28 ± 0.37	24.37 ± 0.79	-	24.36 ± 0.38	24.04 ± 0.70	-

**Systolic BP**	133.06 ± 2.10	134.07 ± 4.48	-	132.41 ± 2.17	136.85 ± 3.72	-

**Diastolic BP**	77.97 ± 0.95	73.89 ± 1.67	-	77.24 ± 0.94	77.04 ± 1.97	-

**Total-C**	6.58 ± 0.11	6.62 ± 0.30	-	6.62 ± 0.12	6.45 ± 0.22	-

**Glucose**	4.76 ± 0.09	5.60 ± 0.5	p = .0120	4.83 ± 0.13	5.30 ± 0.30	-

**LDL-C**	3.06 ± 0.08	3.20 ± 0.23	-	3.08 ± 0.09	3.14 ± 0.15	-

**HDL-C**	1.38 ± 0.04	1.22 ± 0.08	-	1.38 ± 0.04	1.24 ± 0.08	

**Apo B/Apo A**	0.59 ± 0.02	0.71 ± 0.05	p = .0035	0.60 ± 0.02	0.67 ± 0.04	-

**LDL/HDL**	2.43 ± 0.11	2.98 ± 0.28	p = .0389	2.46 ± 0.11	2.87 ± 0.25	-

**Figure 3:**
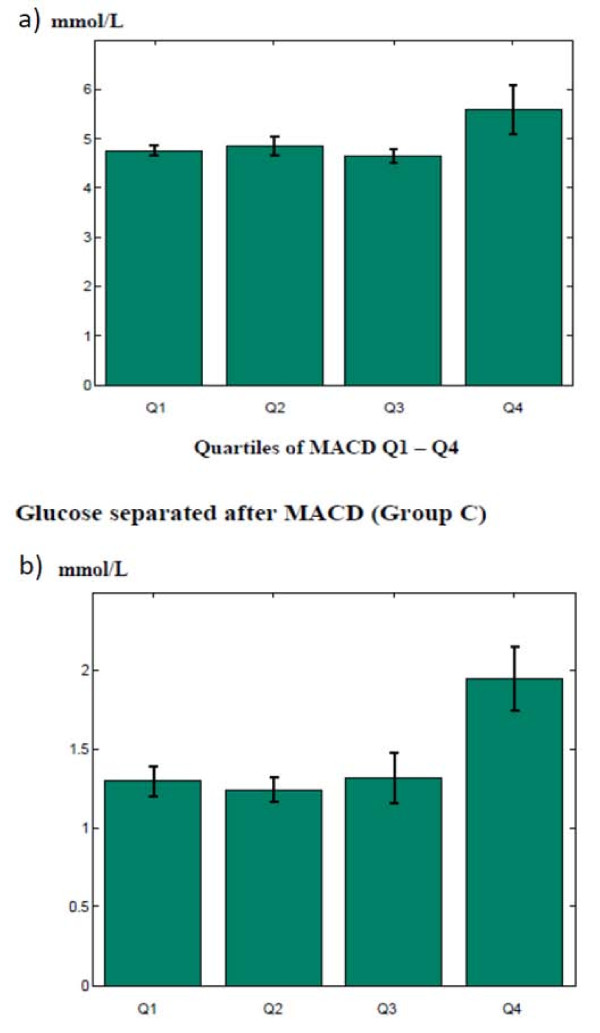
**Glucose level (a) and triglyceride level (b) for the baseline-calcified population in quartiles according to MACD**.

The baseline MACD index was significantly positively associated to baseline blood glucose levels and triglycerides, ApoB/ApoA, and LDL/HDL ratio whereas the AC24 score was not (Table 3). A detailed overview of glucose levels and triglycerides separated after MACD in quartiles is given in figure 3. In the cross-sectional follow-up analysis (Table 4) significance disappeared except for plasma glucose levels, which now was also positively associated to AC24. No significant associations were observed for the cholesterol levels.

**Table 4:** Cross-sectional analysis - division of different biological risk factors into quartiles based on AC24; Here presented mean plus/minus standard error of the mean and results of a two-sample t-test between the highest quartile and the combination of the lowest three quartiles

Follow-Up- Follow-Up	FU-MACD Group SC Lowest three quartiles n = 106	FU-MACD Group SC Highest quartile n = 29	FU-MACD Group SC p-value	FU-AC24 Group SC Lowest three quartiles n = 106	FU-AC24 Group SC Highest quartile n = 29	FU-AC24 Group SC p-value
**Age**	68.98 ± 0.67	73.54 ± 1.28	p = .0019	69.43 ± 0.69	72.18 ± 1.32	-

**Triglyceride**	1.43 ± 0.07	1.45 ± 0.11	-	1.45 ± 0.07	1.39 ± 0.12	-

**BMI**	25.47 ± 0.45	24.60 ± 0.77	-	25.33 ± 0.43	25.21 ± 0.97	-

**Systolic BP**	155.59 ± 2.71	160.41 ± 4.76	-	153.88 ± 2.47	168.50 ± 5.87	p = .0111

**Diastolic BP**	83.51 ± 1.09	81.97 ± 2.29	-	82.62 ± 1.07	85.79 ± 2.38	-

**Total-C**	6.11 ± 0.11	6.24 ± 0.22	-	6.14 ± 0.12	6.13 ± 0.18	-

**Glucose**	5.50 ± 0.10	6.17 ± 0.31	p = .0100	5.51 ± 0.10	6.17 ± 0.32	p = .0148

**LDL-C**	2.88 ± 0.08	3.02 ± 0.17	-	2.91 ± 0.09	2.92 ± 0.15	-

**HDL-C**	1.63 ± 0.04	1.66 ± 0.09	-	1.63 ± 0.04	1.66 ± 0.09	

**Apo B/Apo A**	0.60 ± 0.02	0.61 ± 0.04	-	0.60 ± 0.02	0.62 ± 0.04	-

**LDL/HDL**	1.86 ± 0.07	1.97 ± 0.16	-	1.88 ± 0.07	1.92 ± 0.16	-

Table 5 shows the baseline association of calcification markers after stratification of patients according to standard clinical thresholds. Among the ones investigated, lipids, triglyceride level and LDL level associated positively to both MACD and AC24. Systolic blood pressure showed positive association to both calcification markers.

**Table 5:** Division of MACD and AC24 into halves based on clinical thresholds

Baseline-Baseline	MACD All Smaller	MACD All Larger	MACD All p-value	AC24 All Smaller	AC24 All Larger	AC24 All p-value
**Triglyceride (< 1.69/> = 1.69) 236/60 patients**	60.93 ± 7.21	151.82 ± 28.61	p = .0130	1.51 ± 0.16	2.28 ± 0.38	-

**BMI (< 25/> = 25) 183/125 patients**	80.22 ± 9.75	78.38 ± 14.21	-	1.87 ± 0.19	1.38 ± 0.22	-

**Systolic BP (< 119/> = 119) 104/204 patients**	48.92 ± 11.17	95.05 ± 10.78	p = .0003	0.94 ± 0.19	2.04 ± 0.19	-

**Diastolic BP (< 70/> = 70) 58/250 patients**	64.84 ± 19.04	82.87 ± 9.04	-	1.17 ± 0.29	1.78 ± 0.16	-

**Total Cholesterol (< 5/> = 5) 30/266 patients**	87.77 ± 34.74	78.40 ± 8.53	-	1.63 ± 0.51	1.67 ± 0.15	-

**Glucose (< 5/> = 5) 219/77 patients**	68.76 ± 8.42	109.48 ± 21.48	-	1.54 ± 0.17	2.04 ± 0.33	-

**LDL Cholesterol (< 2.6/> = 2.6) 114/184 patients**	56.95 ± 11.41	93.50 ± 11.47	p = .0071	1.05 ± 0.19	2.04 ± 0.21	p = .0019

**HDL Cholesterol (< 1.55/> = 1.55) 212/86 patients**	89.06 ± 10.92	56.00 ± 10.48	-	1.83 ± 0.18	1.26 ± 0.23	-

Mean plus/minus standard error of the mean and results of a Mann-Whitney U-test between the halves made of baseline MACD and AC24

### Longitudinal analysis - Progression of disease and initiation of disease was analyzed

Table 6 and 7 show the association of follow-up (FU) MACD and AC24 to the different biological parameters. Among all patients progressing in the disease irrespectively of disease status (SC group (n = 135)) the FU-MACD associated significantly with blood lipids (triglyceride, total cholesterol, and LDL), whereas AC24 did not associate to any of the lipid parameters. Both FU-MACD and FU-AC24 were significantly positively associated to systolic blood pressure. Among patients with disease progression (group S (n = 103)) the same pattern of associations was discovered.

**Table 6:** Longitudinal analysis - division of different biological risk factors into quartiles based on AC24

Follow Up-Baseline	FU-MACD Group SC Lowest three quartiles n = 106	FU-MACD Group SC Highest quartile n = 29	FU-MACD Group SC p-value	FU-AC24 Group SC Lowest three quartiles n = 106	FU-AC24 Group SC Highest quartile n = 29	FU-AC24 Group SC p-value
**Age**	60.21 ± 0.67	65.23 ± 1.3	p = .0007	60.69 ± 0.69	63.77 ± 1.33	p = .0433

**Triglyceride**	1.24 ± 0.05	1.51 ± 0.13	p = .0439	1.30 ± 0.06	1.28 ± 0.11	-

**BMI**	24.48 ± 0.40	24.01 ± 0.58	-	24.44 ± 0.36	24.23 ± 0.90	-

**Systolic BP**	125.28 ± 1.78	139.48 ± 4.60	p = .0008	126.08 ± 1.94	137.86 ± 3.84	p = .0065

**Diastolic BP**	76.23 ± 1.00	77.59 ± 2.03	-	76.42 ± 1.02	77.14 ± 2.02	-

**Total-C**	6.39 ± 0.11	7.14 ± 0.25	p = .0048	6.52 ± 0.12	6.63 ± 0.21	-

**Glucose**	4.74 ± 0.09	4.90 ± 0.30	-	4.72 ± 0.09	4.99 ± 0.30	-

**LDL-C**	2.96 ± 0.08	3.42 ± 0.19	p = .0197	3.02 ± 0.08	3.23 ± 0.16	-

**HDL-C**	1.33 ± 0.03	1.34 ± 0.08	-	1.34 ± 0.03	1.29 ± 0.08	-

**Apo B/Apo A**	0.59 ± 0.02	0.66 ± 0.05	-	0.59 ± 0.02	0.63 ± 0.04	-

**LDL/HDL**	2.44 ± 0.11	2.84 ± 0.26	-	2.46 ± 0.12	2.77 ± 0.22	-

Mean plus/minus standard error of the mean and results of a two-sample t-test between the highest quartile and the combination of the lowest three quartiles with baseline biochemistry and baseline X-rays for the SC group follow-up biological risk factors

**Table 7:** Longitudinal analysis - division of different biological risk factors into quartiles based on AC24

Follow Up-Baseline	FU-MACD Group S Lowest three quartiles n = 76	FU-MACD Group S Highest quartile n = 27	FU-MACD Group S p-value	FU-AC24 Group S Lowest three quartiles n = 76	FU-AC24 Group S Highest quartile n = 27	FU-AC24 Group S p-value
**Age**	61.37 ± 0.76	65.24 ± 1.40	p = .0126	61.79 ± 0.80	64.06 ± 1.34	-

**Triglyceride**	1.22 ± 0.06	1.51 ± 0.14	p = .0421	1.29 ± 0.07	1.29 ± 0.12	-

**BMI**	24.21 ± 0.49	23.76 ± 0.60	-	24.10 ± 0.43	24.07 ± 0.92	-

**Systolic BP**	128.55 ± 2.11	139.07 ± 4.83	p = .0231	128.95 ± 2.34	137.96 ± 3.98	-

**Diastolic BP**	77.17 ± 1.12	77.22 ± 2.16	-	77.24 ± 1.14	77.04 ± 2.09	-

**Total-C**	6.36 ± 0.13	7.10 ± 0.27	p = .0124	6.53 ± 0.15	6.60 ± 0.21	-

**Glucose**	4.82 ± 0.12	4.89 ± 0.32	-	4.78 ± 0.12	4.99 ± 0.31	-

**LDL-C**	2.98 ± 0.09	3.38 ± 0.21	p = .0633	3.03 ± 0.10	3.23 ± 0.17	-

**HDL-C**	1.34 ± 0.04	1.33 ± 0.08	-	1.37 ± 0.04	1.27 ± 0.08	-

**Apo B/Apo A**	0.59 ± 0.02	0.66 ± 0.05	-	0.59 ± 0.02	0.64 ± 0.04	-

**LDL/HDL**	2.43 ± 0.13	2.84 ± 0.28	-	2.43 ± 0.14	2.81 ± 0.23	-

Mean plus/minus standard error of the mean and results of a two-sample t-test between the highest quartile and the combination of the lowest three quartiles with baseline biochemistry and baseline X-rays for the S group follow-up biological risk factors.

Assessment of indexes which might be associated with the initiation of aortic calcification (group S^+^, the group with no calcification at baseline (n = 32)) indicated that neither the MACD index nor AC24 did show any significant association to any of the biological parameters at follow-up.

## Discussion

The newly established MACD index was recently shown to be four-fold superior to conventional risk assessment scoring systems, in predicting the CVD related deaths [18]. We examined associations of calcification image biomarkers with other risk factors in order to investigate whether specific biological blood profiles were particularly associated with the MACD index compared to the conventional AC24 score.

The main findings in the cross-sectional study (Table 3) were the highly significant correlation of the MACD index to glucose and triglyceride levels as well as ApoB/ApoA and LDL/HDL ratio compared to that of the AC24 score that did not display any correlation to the two parameters. Furthermore, cross-sectional analysis of the follow-up data displayed a significant correlation to the plasma glucose levels for both the MACD index and the AC24 score emphasizing the importance of glucose for disease progression. The data from the cross-sectional part of the study suggests that an index based on plaque distribution and morphology, such as the MACD index, contains additional information, in terms of its dynamic sensitivity to traditional risk factors.

The longitudinal analysis was performed in order to assess if there were differences in the biological profiles of patients with and without CVD at baseline in relation to the MACD index and AC24 score. There was a large difference in the significance of biochemical factors in the groups with and without calcification at baseline (Table1). When analyzing the group of patients with the increased number of calcifications (SC group n = 135) in their lumbar aorta from baseline to follow-up (Table 6 and 7), we found that the MACD index was highly associated with biochemical parameters such as triglycerides, total cholesterol and LDL cholesterol compared to the AC24 score. The MACD index and the AC24 score both correlated with systolic blood pressure. The longitudinal study indicated that the MACD is a more sensitive index in comparison to the AC24 score, when evaluating the CVD risk in patients having arterial calcifications.

Surprisingly, this picture changes when examining the group of patients with detectable calcifications at follow-up, but not at baseline (S^+ ^group n = 32). This group of patients was used under the current analysis to investigate the early stages in initiation of calcifications. Neither the MACD nor the AC24 score displayed any significant correlation to the biochemical parameters investigated. An obvious caveat is the smaller number of patients (n = 32) in this sub-group compared to the patients progressing in the disease (n = 103).

### MACD index vs. AC24 score

The observed differences in the MACD index and AC24 score in correlation to biochemical factors in the examined sub-groups may be explained by the differences in the scoring technique. As visualized in Figure 1, there are important differences between the AC24 and MACD indexes. The MACD score gives importance to each of the calcified deposits present, whereas only those deposits that are positioned in non-scored areas are included in the AC24 index. For example, the AC24 score doesn't counts two calcified deposits positioned right next to each, but looks only at their projection to the wall (see Figure 1, middle). Thereby the MACD is influenced to a greater degree by an increased number of calcified plaques. In addition, the MACD index favors smaller plaques compared to larger plaques for their potential to grow. This is done by dividing the simulated plaque area estimating the total atherosclerotic area from the visualized calcified core by the actual calcified core area. These subtle although important differences in the two indexes may have significant biological implications, for factors initiating and/or driving the disease. A possible caveat of this specialized feature may be the more tailored performance for long follow-up periods.

The MACD index may have different distribution of plaque number and morphologies that result in the same score. For example, many smaller plaques would have a higher score compared to an equal area of large plaques. Thereby the same amounts of calcification assessed by the MACD score may change significantly over time, both resulting in lower and higher risk. Thereby the MACD index may be a more dynamic assessment of score and risk. In support of this, recent investigation suggests that calcium content of the plaque may be proportional to the risk of rupture until a certain point, at which the evolving plaque becomes a more stable plaque [18]. This biological feature is accounted for in the modulation of the potential to grow of smaller plaques.

### Plasma Glucose and CVD-risk

One of our main findings at baseline demonstrated a strong correlation of high plasma glucose levels with MACD but not with the AC24 index. At follow up, cross-sectional analysis, we found significant association of both MACD index and AC24 score with glucose levels (Table 3 and 4, and figure 3a). This suggests a strong predisposing role of glucose in atherogenesis. Diabetes is now generally considered being one of the major risk factors of atherosclerosis, with the hyperglycemia as an underlying contributing factor [22]. Patients with diabetes have been found to present with distinct calcifications of the medial part of the arteries often referred to as Monckenberg's medial sclerosis, which is associated with vascular stiffening and atherosclerosis [23-26]. Intimal calcifications may occur independently of medial calcifications and *vice versa *[27]. In diabetic patients, medial calcification seems to be a strong independent predictor of cardiovascular mortality and occurs particularly in patients with nephropathy [28,29]. However, mechanisms that mediate vascular complications following hyperglycemia are not yet fully understood.

### Blood-lipids and CVD-risk

Our second major cross-sectional finding demonstrates that plasma triglyceride levels correlated with the MACD index in the baseline cross-sectional study (Table 3 and 4 and figure 3b), adding to the body of evidence that triglycerides are of considerable importance in atherogenesis.

Elevated serum triglycerides in the high-risk patients and the role of hypertriglyceridemia as causality of coronary artery disease remain to be elucidated. Nonetheless, there is growing evidence that hypertriglyceridemia is a marker for increased risk for coronary artery disease [30-33]. Our data further support this notion, both in the cross-sectional and the longitudinal part of the study. Importantly, the triglycerides correlate significantly the MACD-index, but not AC24, emphasizing the importance and differences of this new scoring method.

It should also be emphasized that the observed strong baseline correlation of blood lipids, disappeared in the follow-up cross-sectional study. We have previously shown [18] that the high-risk group of patients in our cohort presented with OR of approximately 20, according to the MACD index. The explanation for the slightly improved blood-lipid picture at follow-up could partly be due to the death of some of the patients in this group during the 7.5 years between baseline and follow-up visit. Furthermore, as the age in the population increases, the treatments in form of blood-lipid lowering medication increases in our population, which could contribute to the understanding of the sudden lack of significant correlations of both the MACD index and the AC24 score to blood lipids. We do not have information on the number of patients receiving lipid-lowering medication in our study population.

### Limitations of the Study

The sample size is a limitation of the present study. The relatively small population with only 20 CVD deaths, a limited representation of ethnicity and gender and a mixture of death causes may limit the generalizability of our results. Furthermore, the lack of information about lipid lowering treatments is a problem of a historical study.

## Conclusion

In conclusion, we have demonstrated that a refined index of arterial aortic calcification, MACD, may contain additional and important information on risk related to cardiovascular mortality compared to the traditional AC24 index. This index correlated significantly to emerging risk factors in atherosclerosis. Further studies in larger cohorts are needed to further differentiate and understand the relative importance of MACD index compared to that of the AC24 score. In the present studies, each index provided important information. However, whereas the MACD index correlated to both initiation and progression of disease, the AC24 correlated only in relation to initiation of disease.

### Study limitations

The present study has its limitations. Its findings are only valid for a follow-up period of 8.5 years and may not necessarily apply to shorter follow-up periods. For short follow-up times, the predictive power could possibly be based only on the total plaque burden as described by the AC24 score. Furthermore, the present population is restricted in size, geographical and ethnic content to postmenopausal Danish women. The present study needs validation in other populations and longer term clinical settings, preferably supported by the histological evaluations.

## Competing interests

Morten A Karsdal is the CEO of the company. Other authors declare that they have no competing interests.

## Authors' contributions

NB - first author, manuscript preparation, data analysis, pathophysiological discussions

MG - first author, manuscript preparation data acquisition and analysis, mathematic models

MN - manuscript drafting, data acquisition, data analysis and interpretation

TCR - manuscript drafting, data interpretation

LMR - manuscript drafting, data interpretation

MAK - manuscript drafting, data analysis and interpretation

CC - manuscript drafting, data analysis and interpretation

All authors have read and approved the final manuscript.

## Pre-publication history

The pre-publication history for this paper can be accessed here:

http://www.biomedcentral.com/1471-2261/11/75/prepub
